# WOA-Based Robust Congestion Control Scheme with Two Kinds of Propagation Latencies and External Disturbance in Software-Defined Wireless Networks

**DOI:** 10.3390/biomimetics8020249

**Published:** 2023-06-10

**Authors:** Xi Hu, Zhiwei Shen, Xin Xiong, Siqi Zhang, Junming Chang, Wang Gao

**Affiliations:** 1 State Key Laboratory of Precision Blasting, Jianghan University, Wuhan 430056, China; huxi027@163.com; 2 School of Artificial Intelligence, Jianghan University, Wuhan 430056, China; sinkia0723@gmail.com (S.Z.); cjm72@163.com (J.C.); gaow@jhun.edu.cn (W.G.); 3 Artificial Intelligence Institute, Jianghan University, Wuhan 430056, China; 4 Wuhan Chaoxing Digital Library Education Technology Co., Ltd., Wuhan 430050, China; wwlszw@foxmail.com

**Keywords:** AIMD adjustment scheme, Lyapunov–Krasovskii functionals, robust congestion control scheme, SDWN, two kinds of propagation latencies and external disturbance, WOA algorithm

## Abstract

This paper proposes a novel WOA-based robust control scheme with two kinds of propagation latencies and external disturbance implemented in Software-Defined Wireless Networks (SDWNs) to maximize overall throughput and enhance the stability of the global network. Firstly, an adjustment model developed using the Additive-Increase Multiplicative-Decrease (AIMD) adjustment scheme with propagation latency in device-to-device paths and a closed-loop congestion control model with propagation latency in device–controller pairs are proposed, and the effect of channel competition from neighboring forwarding devices is analyzed. Subsequently, a robust congestion control model with two kinds of propagation latencies and external disturbance is established. Then, a new WOA-based scheduling strategy that considers each individual whale as a specific scheduling plan to allocate appropriate sending rates at the source side is presented to maximize the global network throughput. Afterward, the sufficient conditions are derived using Lyapunov–Krasovskii functionals and formulated using Linear Matrix Inequalities (LMIs). Finally, a numerical simulation is conducted to verify the effectiveness of this proposed scheme.

## 1. Introduction

With the rapid development of large-scale network deployments, traditional wireless networks, such as Wi-Fi or cellular networks, have adopted a distributed architecture, where network intelligence is embedded in each access point or base station. This makes it difficult to manage and configure a network as a whole, especially in large-scale deployments. Software-Defined Networking (SDN) is an architectural approach that separates the control plane and data plane in network devices, thereby enabling centralized control and management of the network through a programmable software controller [1,2,3,4,5]. Software-Defined Wireless Networking (SDWN) is a concept that extends the principles of SDNs to wireless communication networks, which apply these principles to wireless networks, enabling greater flexibility, scalability, and control over wireless infrastructure.

In SDWNs, similar to SDN, the control plane is separated from the data plane [1,2,3,4,5,6,7,8]. The control plane consists of a centralized software controller that manages and orchestrates the network resources, makes decisions about network policies and configurations, and dynamically controls the behavior of the wireless network infrastructure. The data plane comprises the wireless access points or base stations that handle the transmission and reception of data. The control plane of each forwarding device can only operate correctly when connected to SDWN-centralized controllers [1,2,3,4,5,6,7,8,9].

With the continuous expansion of the scale of networks and user populations, an excessive number of network services may cause network congestion in SDWNs [10,11], which has provoked research into effectively controlling network congestion and stabilizing networks [12,13]. Congestion control is a crucial technology used in wireless networks to manage and prevent network congestion, employing congestion control mechanisms to regulate the sending rate when there is a high demand for wireless network resources that exceeds the available capacity. Congestion control technologies play a vital role in maintaining the stability, efficiency, and performance of wireless networks, thus ensuring that wireless network resources are utilized optimally. Stability congestion control, which is intended to maximize the throughput and enhance the stability of global networks, has drawn widespread attention and research interest [14,15]. Propagation latency and external disturbance are often considered to constitute two critical factors that affect global network stability. Propagation latency may lead to additional network costs and unreliability [16], while the variability of external disturbance based on the relevant wireless characteristics leads to abrupt structural variations [17]. Even if a wireless network is stable via stability congestion control, the global network may become unstable again because of these two factors and may not be able to maintain long-term stability.

Therefore, it is essential to restabilize SDWNs’ network parameters with optimal values to maintain long-term stability. Robust control, which maintains a global network under long-term control with propagation latency and external disturbance, is likely a solution with which to tackle this re-stabilization problem. Thus, robust congestion control is defined as a robust control scheme acting on network congestion in order to enable higher network efficiency and lower network congestion.

Some existing solutions favor the adoption of traditional network control methods to analyze the congestion control problem in SDWNs. A traditional network control system is modeled using stochastic network-induced latency [18], in which the analytical study of network stability has been implemented to solve network-induced latency and design feedback control algorithms. In the SDWN architecture, a global network controller is responsible for the rate management of each OpenFlow device [1,2,19,20,21,22,23]. In the separation of the control and data planes, control plane unification is implemented for different kinds of networks, including wired Internet Protocol (IP) networks [5,6,8,20,23], Wavelength-Division Multiplexings (WDMs) [24], and wireless networks [25,26,27]. In recently published works, the AIMD adjustment scheme has been further optimized using Lyapunov–Krasovskii functionals for network congestion control in SDWNs [28,29]. In terms of the congestion control framework, the Additive-Increase Multiplicative-Decrease (AIMD) adjustment scheme is used to tackle network congestion control problems through the execution of proper source adjustments in SDWNs [30,31,32]. In terms of robust control algorithms, various forwarding information control algorithms have been proposed through analyses of SDWN-centralized controllers with respect to improving network robustness and reacting to failures [2,23,33,34,35]. In [36,37,38], robust congestion control schemes have been proposed in order to achieve maximal network throughput in device-to-device paths by using Lyapunov–Krasovskii functionals. In terms of meta-heuristic optimization algorithms, several meta-heuristic optimization algorithms, such as the Social Spider Optimization (SSO) algorithm [39], Bat Algorithm [40], and Particle Swarm Optimization (PSO) algorithm [41], have been utilized in wireless networks to greatly reduce the number of network data required to solve the network congestion problem [42]. In this paper, we use the Whale Optimization Algorithm (WOA) to more effectively achieve optimization objectives using a new global scheduling strategy for solving the network congestion problem. The WOA algorithm, which simulates the foraging behavior of humpback whales, was first proposed in [43]. It is often used to find the optimal solution for global optimization problems in various fields, among which those covered in reviews include engineering, clustering, classification, robot paths, image processing, networks, task scheduling, and other engineering applications [44,45,46,47,48].

However, these solutions are limited by the following aspects.

(i) The control laws are separated from the centralized controllers but integrated into the forwarding devices in the form of flow tables;
(ii) An SDWN architecture with two kinds of propagation latency is seldom considered for robust congestion control;
(iii) The traditional theories are not compatible with the robust congestion control theory pertaining to SDWNs.

The major contributions of this paper are summarized as follows:

(i) First, we provide a novel sending rate adjustment model with propagation latency in device-to-device paths as the fundamental model in the forwarding layer. Then, we establish a closed-loop congestion control model with propagation latency in device–controller pairs as a supplementary model. The propagation latency strengthens the veracity of the stability analysis, and its influence is considered in both device-to-device paths and device–controller pairs from a global perspective. Moreover, we consider channel competition near the forwarding devices in order to more effectively design the congestion control model based on the broadcasting nature of the wireless medium.

(ii) We design a new, weighted, fair scheduling strategy to pre-set the control objective of the stability congestion control scheme in order to solve the global robust congestion problems faced by SDWNs, which is utilized to calculate the optimized values of network parameters. Moreover, external disturbance is also considered for robust congestion control. To eliminate external disturbance from the global network system, the stability congestion control model had to be converted into a robust congestion control model; consequently, the optimized status was maintained via the transformation of two closed-loop congestion control models into a normal robust H∞ control model.

(iii) An interdisciplinary effort is made to construct a robust congestion control scheme by combining stability analysis theory and congestion control principles in SDWNs. Exploiting the applicability of Lyapunov–Krasovskii functionals in stability analysis, this paper constructs novel optimized Lyapunov–Krasovskii functionals acting on the robust H∞ control model to achieve the desired global robust control system for solving network congestion.

(iv) We design experiments on both the variations of error states and the energy trajectories for a robust H∞ control scheme implemented in the SDWN, including the AIMD adjustment scheme and the information-forwarding and control algorithm acting as benchmark algorithms. To evaluate the control performance, ablation experiments were conducted, which demonstrated the superiority of our proposed robust control scheme over the AIMD adjustment scheme and the information-forwarding and control algorithm with respect to maximizing the global SDWN throughput to maintain long-term stability with two kinds of propagation latencies and external disturbance.

In SDWNs, the forwarding devices first record their congestion state information and explicitly advertise it to the centralized controllers. The centralized controllers provide some control policies and send control instructions to the forwarding devices. Next, the forwarding devices follow these control instructions and make proper adjustments to the sending rates at the source-side. Previous works, such as [36,37,38], studied the robust congestion control scheme with propagation latency in device–controller pairs, while this paper defines the propagation latency in both device–controller pairs and device-to-device paths as an upper bound of latency. Thus, two closed-loop congestion control models are established for the further research of the robust congestion control scheme. In our study, the AIMD adjustment scheme is still initially adopted to analyze network congestion with propagation latency in device–controller pairs, and two basic congestion control models are established. Next, a novel WOA-based scheduling strategy that considers each individual whale as a specific scheduling plan to allocate appropriate sending rates at the source side is proposed in the SDWN-centralized controllers to make proper adjustments in each forwarding device. Then, a novel robust congestion control model is proposed through the use of Lyapunov–Krasovskii functionals [49,50], and a theorem is proposed to determine the sufficient conditions for the robust control. These sufficient conditions are expressed as Linear Matrix Inequalities (LMIs). Finally, numerical instances are provided to demonstrate the effectiveness of our proposed scheme, which is able to more realistically analyze robust congestion control schemes under the influence of propagation latencies and external disturbance, over traditional schemes and those from previous works.

The following are also discussed in the remaining sections of this paper. Section 2 presents a brief overview of related works. In Section 3, an analytical network model, which was developed by implementing an AIMD adjustment scheme, is established to adjust the sending rate at the source side, and a WOA-based scheduling strategy that considers each individual whale as a specific scheduling plan to allocate appropriate sending rates at the source side is presented to address the error states of the sending rate. Section 4 proposes a robust congestion control problem formulation, and some preliminaries are introduced. Section 5 addresses network congestion control by using Lyapunov–Krasovskii functionals and calculates sufficient conditions. Section 6 reports the results of a numerical network simulation to demonstrate the effectiveness of our proposed robust congestion control scheme, and comparisons with other congestion control approaches applied in SDWNs are also provided. Section 7 presents the conclusions and directions for future work.

## 2. Related Works and Problem Motivation

### 2.1. Related Works

SDN is a network paradigm separating the control and data planes of a network. The OpenFlow protocol was first introduced at Stanford University in 2008 [1]. Since then, SDN controllers and the OpenFlow protocol have been advanced to improve network performance [2,3,4,5,6]. SDWN has applications in various domains, including enterprise networks, campus environments, public Wi-Fi facilitation, Internet of Things (IoT) networks, and 5G cellular networks. By leveraging the principles of SDN, SDWN offers a more flexible, manageable, and scalable approach to wireless network management, thereby paving the way for innovation and improved performance in wireless communications [5,6,7,8,9].

To solve the network congestion problem experienced by SDWNs, the traditional robust control methods rely on either an AIMD adjustment scheme or an information-forwarding and control algorithm.

#### 2.1.1. AIMD Adjustment Scheme

As a traditional form of network control, the AIMD adjustment scheme is often employed at the source side. It has been researched for decades, and many reliable robust control solutions have been proposed for SDWNs. By utilizing improved AIMD adjustment schemes and queue congestion management, the author of [30] proposed an SDN-based Explicit-Deadline-Aware Transmission Control Protocol (TCP) mechanism for cloud data center networks. In [31], the authors adopted the AIMD adjustment scheme and introduced an Additive-Decrease Multiplicative-Increase (ADMI) approach to preserve bandwidth. The author of [30] proposed a load balancer application using an AIMD adjustment scheme based on various features of SDN and OpenFlow. In recently published works, Lyapunov–Krasovskii functionals were utilized, along with the AIMD adjustment scheme, for network congestion control in SDNs. In [28], the authors modeled a multi-objective minimization allocation scheme of energy and delay using traditional Lyapunov–Krasovskii functionals. The authors of [29] proposed a queueing model and solved a problem regarding long-term network utility maximization using traditional Lyapunov–Krasovskii functionals. However, the key limitation of these approaches is that congestion control was implemented at the source side, which does not allow for the achievement of network robustness with a global view of SDWN.

#### 2.1.2. Information-Forwarding and Control Algorithm

The SDWN architecture provides logically centralized controllers for receiving updates and implementing control policies. Most of the current reliable robust control solutions are information-forwarding and control algorithms. In [2], Vissicchio and Cittadini introduced an operational sequences computation algorithm to compute operational sequences that preserves the correctness of forwarding and policies to ensure the robustness of SDN updates. During the update, this algorithm robustly implements unpredictable factors, such as delayed message delivery and processing. In [23], the authors proposed a distributed OpenFlow-based routing protocol to improve network robustness, reaction to failures, and controller scalability. This protocol can provide robustness for topological failures and rapidly reduce the path stretch. The authors of [31] presented a congestion-aware and robust, reliable multicast method for small groups in data centers that can dynamically bypass congested and failing links and then achieve high efficiency and robustness. In [34], the author analyzed the requirements of a secure, robust, and resilient controller for providing security improvements. The authors of [35] proposed a fault-prone, concurrent control scheme for robust policy implementation in distributed SDNs. The authors of [36,37,38] proposed robust congestion control schemes for achieving maximal network throughput by considering the propagation latency in device-to-device paths.

However, propagation latency, especially in device–controller pairs, must be further considered and discussed as a key factor for robust congestion control in SDWNs. Simultaneously, external disturbance needs to be analyzed as the other key factor. Moreover, previous approaches to addressing network congestion implemented the robust control of partial networks instead of considering a global view of SDWN.

Despite the abundant literature on SDWNs, both approaches are limited in terms of implementing global robust congestion control in the presence of propagation latency and external disturbance.

### 2.2. Motivation

Due to an SDWN’s characteristics, its wireless environment is susceptible to network congestion due to its limited available spectrum and bandwidth, shared medium, and varying channel conditions. SDWNs have finite bandwidth, in which network congestion can significantly impact data rates and throughput. By effectively controlling network congestion in SDWNs, bandwidth utilization can be optimized by allocating the network resources reasonably in order to facilitate the fair sharing of available bandwidth between all the devices. Moreover, the network congestion in SDWNs may lead to wasteful retransmissions and inefficient utilization of all network resources. By mitigating congestion in SDWNs, our method can reduce unnecessary retransmissions and improve overall resource efficiency, thereby enhancing network sustainability.

Therefore, this paper focuses on solving the re-stabilization problem pertaining to a typical SDWN architecture with two kinds of propagation latency and external disturbance via the robust congestion control scheme, aiming to maximize global network throughput and re-stabilize network parameters at their optimal values. This can better address the network congestion problem in SDWNs, which is essential to ensure the network’s optimal performance and reliability. By leveraging an SDWN’s centralized control and dynamic management capabilities, network congestion can be proactively managed, and network resources can be reasonably allocated to enhance the efficiency and effectiveness of SDWN operations.

(i) To maximize the global SDWN throughput, this paper presents a novel WOA-based scheduling strategy that considers each individual whale as a specific scheduling plan in order to pre-set the network parameters at optimal values. The sending rate at the source side, constituting a key network parameter, is the target of the robust congestion control scheme for network congestion control.

(ii) To re-stabilize the network parameters at their optimal values, the differences between the current and optimized states are first assigned to the error states. Next, the robust congestion control problem with propagation latency and external disturbance is viewed as a robust control problem. Finally, the robust control problem based on the error states is addressed by referencing Lyapunov–Krasovskii functionals.

## 3. Model and Analysis

**Figure 1:**
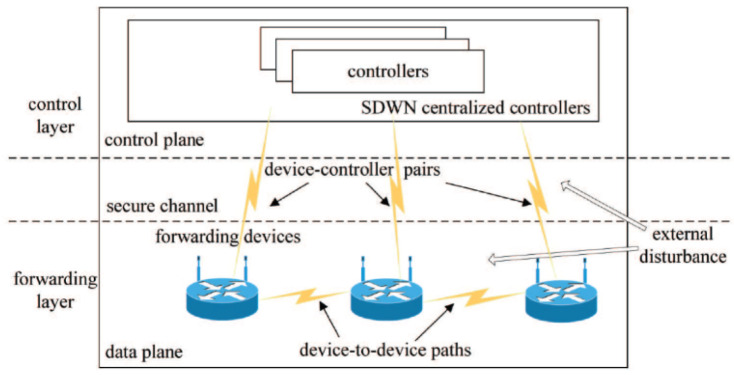
A typical SDWN architecture with two kinds of propagation latency and external disturbance.

A typical SDWN architecture with two kinds of propagation latency is presented in Figure 1. Two kinds of propagation latency exist in SDWN: (1) propagation latency in the device-to-device path, and (2) propagation latency in device–controller pairs, which divides the entirety of SDWN into two closed-loop networks for analyzing network congestion. SDWN-centralized controllers consist of a series of controllers with distributed designs for enhancing network reliability, scalability, and resiliency [35]. The OpenFlow-based forwarding devices at the source side advertise their state information to the centralized controllers via a wireless channel and properly adjust their sending rates via the AIMD adjustment scheme after receiving the control instructions from the centralized controllers. These control instructions are provided to process individual network services by means of the WOA-based scheduling strategy. In order to maintain the network parameters’ long-term stability in the SDWN, this paper focuses on maximizing the global SDWN throughput and stabilizing global network parameters at their optimal values under the robust congestion control scheme with two kinds of propagation latency and external disturbance. Thus, a new WOA-based scheduling strategy that considers each individual whale as a specific scheduling plan to allocate appropriate sending rates at the source side is adopted to optimize global network performance by properly arranging the network parameters. There is an optimized stable state constituting a key network parameter in each forwarding device for the robust congestion control scheme.

The analysis of these two closed-loop congestion control systems is classified into four parts in the subsections below.

### 3.1. A Sending Rate Adjustment Model with Propagation Latency in Device-to-Device Paths

First, in order to analyze the sending rate adjustment at the source side, the following assumptions and definitions are proposed.

**Assumption** **1.** *There exist infinite flows at the source side that await transmission.*

**Definition** **1.** There exists an ideal queue length $x_{l}^{*}$ that has been verified as being capable of achieving the best performance with respect to the sending rate arrangement after multiple experiments.

**Assumption** **2.** Define two queue lengths in any forwarding device, where one is the current queue length $x_{l}$ and the other is the ideal queue length $x_{l}^{*}$. At the source side, the variation in the sending rate is represented as the difference of the queue length ${\bar{x}}_{l},{\bar{x}}_{l}=x_{l}-x_{l}^{*}$. If the difference value ${\bar{x}}_{l}>0$, the sending rate additively increases; otherwise, when ${\bar{x}}_{l}<0$, the sending rate multiplicatively decreases. The magnitude of the difference value positively correlates with the level of rate variation at the source side. An AIMD adjustment scheme is implemented in every forwarding device based on the difference value ${\bar{x}}_{l}$.

**Assumption** **3.** *The neighboring forwarding devices record their congestion state information and periodically send them to the SDWN-centralized controllers. Then, the centralized controllers optimize some control laws via the congestion state information and send them to the source-forwarding device according to the control instructions. We assume the existence of a local Congestion State (CS) value that is incorporated into the control instruction. This feedback control instruction shows the CS reflected in the current condition of the neighboring links in the whole round-trip. The CS is either non-positive (${\bar{x}}_{l}>0$, i.e., no congestion occurred) or positive (${\bar{x}}_{l}<0$, i.e., congestion occurred).*

**Figure 2:**
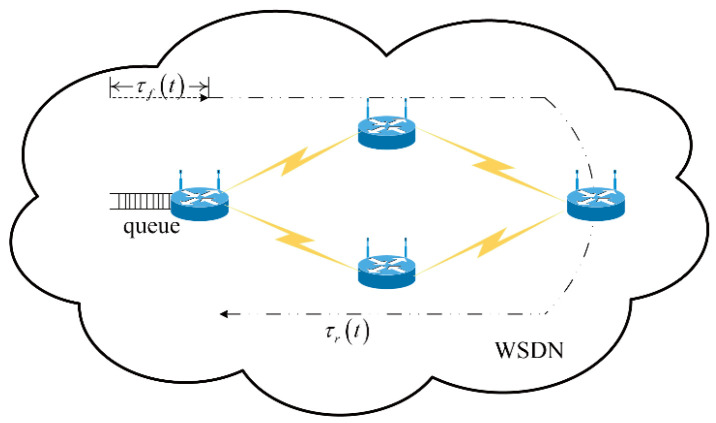
Data transmission process with propagation latency in device-to-device paths when utilizing the AIMD adjustment scheme in SDWNs.

Figure 2 presents an example of the data transmission process with propagation latency in device-to-device paths in the SDWN, which can be modeled as a closed-loop congestion control system. By incorporating the CS feedback from the centralized controllers and using the AIMD adjustment scheme to tackle the congestion control problems, the AIMD parameters are analyzed, and the basic network congestion model is established as a linear continuous closed-loop congestion control system.

In the SDWN, the neighboring forwarding devices shift their congestion state information to the centralized controllers, in which the global network traffic state messages are concentrated. After an essential analysis, the centralized controllers optimize their control laws and make proper adjustments to every sending rate at the source side. If network congestion has occurred in the neighboring forwarding devices, said devices feed control instructions (CS > 0) back to the source forwarding devices within fixed time intervals to communicate the adjustments of the sending rates; when a state of non-congestion occurs in the neighboring forwarding devices, they feed control instructions (CS < 0) back.

At the source side, suppose that the CS occurs at the moment t,t≥0, for which the time-varying sending rate is denoted as $r\left(t_{i}\right)$. $\tau_{r}\left(t\right)$ and $\tau_{f}\left(t\right)$ denote process latency and forward channel propagation latency, respectively. Using the AIMD adjustment scheme, this section considers the fixed constant weight $A_{f}$ as indicating an additive-increase and $D_{f}$ as indicating a multiplicative-decrease, respectively. At the source side, if the CS is non-positive, the sending rate increases by weight $A_{r}$; otherwise, CS is positive, and the sending rate decreases by weight $D_{r}$. Thus, the behavioral equation can be expressed as follows:

$$\begin{array}{ll} r\left(t_{i}\right) & =r\left(t_{i-1}\right)-\{A_{r}\left[1-\eta \left(x_{l}\left(t-\tau_{r}\left(t\right)\right)-x_{l}^{*}\right)\right]\\ & -D_{r}r\left(t\right)\left[\eta \left(x_{l}\left(t-\tau_{r}\left(t\right)\right)-x_{l}^{*}\right)\right]\}, \end{array}$$

Then, we obtain

$$\begin{array}{ll} \frac{r\left(t_{i}\right)-r\left(t_{i-1}\right)}{t_{i}-t_{i-1}} & =\frac{1}{t_{i}-t_{i-1}}\{A_{r}\left[1-\eta \left(x_{l}\left(t-\tau_{r}\left(t\right)\right)-x_{l}^{*}\right)\right]\\ & -D_{r}r\left(t\right)\left[\eta \left(x_{l}\left(t-\tau_{r}\left(t\right)\right)-x_{l}^{*}\right)\right]\}\\ & =-B_{r}r\left(t_{i}-\tau \left(t\right)\right)\{A_{r}\left[1-\eta \left(x_{l}\left(t-\tau_{r}\left(t\right)\right)-x_{l}^{*}\right)\right]\\ & -D_{r}r\left(t\right)\left[\eta \left(x_{l}\left(t-\tau_{r}\left(t\right)\right)-x_{l}^{*}\right)\right]\}, \end{array}$$

where $B_{r}$ is defined as a fixed constant weight, $B_{r}r\left(t_{i}-\tau \left(t\right)\right)=\frac{1}{t_{i}-t_{i-1}}$. η is the sensitivity degree of the adjustment, $\eta \left(x_{l}-x_{l}^{*}\right)$ denotes the probability parameter, $x_{l}\left(t\right)$ represents instantaneous queue length, and t is the current moment.

Let $a=A_{r}B_{r},b=D_{r}B_{r}$, and suppose r = c, $x_{l}=x_{l}^{*}$ in an equilibrium state; thus, the following is yielded:

(1) $$\{\begin{array}{l} \dot{r}\left(t\right)=\left(-ac\eta -bc^{2}\eta \right)\left(x_{l}\left(t-\tau_{r}\left(t\right)\right)\right)-bc\left(\frac{a}{a+bc}r\left(t\right)\right),\\ {\dot{x}}_{l}\left(t\right)=r\left(t-\tau_{f}\left(t\right)\right). \end{array}$$

Eliminating $x_{l}\left(t\right)$ from (1) yields the second-order differential equation

(2) $$\ddot{r}\left(t\right)+\kappa \dot{r}\left(t\right)+\vartheta r\left(t-\tau_{1}\left(t\right)\right)=0,$$

where $\kappa =\frac{abc}{a+bc},\vartheta =c\eta \left(a+bc\right)$ are the parameters of this second-order system, and $\tau_{1}\left(t\right)=\tau_{r}\left(t\right)+\tau_{f}\left(t\right)$ denotes the round-trip of propagation latency from the source forwarding device to the destination. Note that the second-order dynamic Equation (2) can be rewritten in a matrix form, as follows.

(3) $$\dot{r}\left(t\right)=\left(\begin{array}{cc} 0 & 1\\ 0 & -\kappa \end{array}\right)r\left(t\right)+\left(\begin{array}{cc} 0 & 0\\ -\vartheta & 0 \end{array}\right)r\left(t-\tau_{1}\left(t\right)\right).$$

Note that $\hat{A}=\left(\begin{array}{cc} 0 & 1\\ 0 & -\kappa \end{array}\right),\;A_{d}=\left(\begin{array}{cc} 0 & 0\\ -\vartheta & 0 \end{array}\right)$ are the weights of the network parameters. Thus, Equation (3) can be converted into

(4) $$\dot{r}\left(t\right)=\hat{A}r\left(t\right)+A_{d}r\left(t-\tau_{1}\left(t\right)\right).$$

In this section, Equation (4), as the state variable equation, represents a closed-loop congestion control system, which utilizes an AIMD adjustment scheme at the source side after receiving state feedback. Obviously, the congestion control system shown in Figure 2 can be modeled using Equation (4). Solving Equation (4) yields the solution to the congestion control problem.

### 3.2. A Closed-Loop Congestion Control Model with Propagation Latency in Device–Controller Pairs

**Figure 3:**
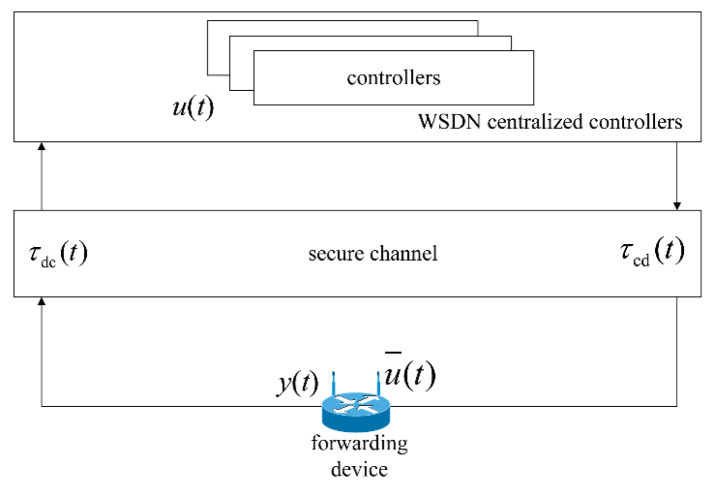
Data transmission process with propagation latency in device–controller pairs in SDWNs.

As shown in Figure 3, the propagation latency from a forwarding device to the centralized controllers (DC) and that from the centralized controllers to a forwarding device (CD) are defined as $\tau_{dc}\left(t\right)$ and $\tau_{cd}\left(t\right)$, respectively. Assume that the centralized controllers can monitor $\tau_{dc}\left(t\right)$ and that the forwarding devices can receive the CS from the centralized controllers with $\tau_{cd}\left(t\right)$. Let $\tau_{2}\left(t\right)=\tau_{dc}\left(t\right)+\tau_{cd}\left(t\right)$, which is termed propagation latency in device–controller pairs.

When a flow joins the SDWN or is generated in an OpenFlow forwarding device, it is first placed in a queue, in which is waits to be processed and sent. When a communication channel is free, the centralized controllers establish a device-to-device path after receiving all communications from the whole network. Then, they design a control policy and sends the control instructions to adjust the sending rate at the source side. The whole process is described as follows.

First, the flow entry in the source forwarding device sends a complete or partial copy of the sending rate r(t) to the centralized controllers (a packet-in message). Next, the centralized controllers calculate the state of the forwarding device by means of the packet-in message, classify the global state information, and create a control policy to stabilize the sending rate. The control policy is utilized to re-stabilize the sending rate via control instructions $\bar{u}\left(t\right)$. Then, the controllers adjust the weighted matrix (matrix $B_{u}$, $B_{u}\in {\mathbb{R}}^{n\times n}$) accordingly, where $B_{u}$ represents the completion of the flow (which generates the packet-in message) associated with the control instructions.

Therefore, the SDWN architecture with propagation latency in device–controller pairs can be modeled as

(5) $$\dot{r}\left(t\right)=\bar{A}r\left(t\right)+B_{u}\bar{u}\left(t\right),$$

where $\bar{A}$ is the matrix of the network parameters.

Let the control instruction $u\left(t\right)=Kr\left(t\right),\;\;K\in {\mathbb{R}}^{n\times n}$ is denote control strength, and the control instruction be represented as $\bar{u}\left(t\right)=Kr\left(t-t_{k}\right),\;t\in \left[t_{k},t_{k+1}\right),$ where $\bar{u}\left(t\right)$ is the control input in the forwarding device, and $t_{k}$ is the sample time at moment k. Rewrite r(t) as $r\left(t_{l}\right)=r\left(t-\left(t-t_{k}\right)\right)=r\left(t-\tau_{2}\left(t\right)\right),\;t\in \left[t_{k},t_{k+1}\right),$ where $\tau_{2}\left(t\right)=\tau_{dc}\left(t\right)+\tau_{cd}\left(t\right)$ denotes the entirety of propagation latency in the device–controller pairs.

Substitute $r\left(t-\tau_{2}\left(t\right)\right)$ into Equation (5); consequently, the SDWN architecture with propagation latency in device–controller pairs becomes a linear closed-loop congestion control system.

(6) $$\begin{array}{l} \dot{r}\left(t\right)=\bar{A}r\left(t\right)+B_{u}\bar{u}\left(t\right)=\bar{A}r\left(t\right)+B_{u}Kr\left(t-\tau_{2}\left(t\right)\right),\\ t\in \left[t_{k},t_{k+1}\right). \end{array}$$

### 3.3. Effect of Channel Competition from Neighboring Forwarding Devices

**Figure 4:**
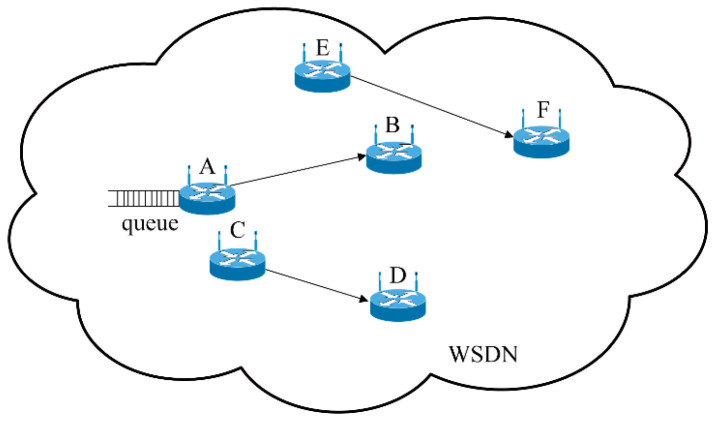
Channel competition between neighboring forwarding devices in SDWNs.

The problem of wireless channel competition is a critical issue impacting the sending rate adjustment at the source side in the SDWN. The centralized controllers contain information on global topology. Due to the broadcasting nature of the wireless medium, the forwarding devices cannot use and occupy the same wireless channel at the same moment. The other forwarding devices around the source forwarding device may contain data that can be transmitted simultaneously. Thus, every forwarding device needs to compete for the shared channel in order to send data, as shown in Figure 4. Optimizing the sending rates of the different source forwarding devices is essential in a period of channel competition. By centralizing control in the SDWN, the effect of coupling connection is reflected in the control instructions received from the centralized controllers. All network information is aggregated, and the control laws are designed in the centralized controllers to control network congestion.

Figure 4 shows an instance of channel competition in the SDWN. When there are data that have been sent from the forwarding device A to the forwarding device B, A provides updated information to the centralized controllers. At this moment, forwarding device C communicates with forwarding device D, occupying the wireless channel. Thus, forwarding device A receives the control instructions and must wait until the wireless channel is free. Meanwhile, when forwarding device E coupled with forwarding device F also have data to transmit, they must also compete with forwarding device A. The centralized controllers require all information and make proper adjustments of the control laws of the different forwarding devices, which feed this information back to the forwarding devices via the control instructions.

**Assumption** **4.** *The coupled forwarding devices are defined as the reciprocal channel effect in SDWNs. The whole network is diffusively coupled, and all network information is sent to the centralized controllers. Define $L={\left(l_{\mathrm{ij}}\right)}_{N\times N}$ as the Laplace coupling matrix, whose diagonal elements are considered to correspond to $l_{ii}=-\Sigma_{j=1,j\neq i}^{N}l_{ij},\;l_{ij}\geq 0$. This represents the network topology of the global SDWN. If there is a connection between forwarding device i and j (i.e., i and j are neighbors), $l_{ij}=l_{ji}=1$; otherwise, $l_{ij}=l_{ji}=0\;\left(i\neq j\right)$. The row sum of L is zero. The whole SDWN is connected, and matrix L is irreducible.*

Based on the Laplace coupling matrix L in Assumption 4, the control policies ${\hat{u}}_{i}\left(t\right)$ in the forwarding device i that represent the topology relationship between all neighbor forwarding devices can be described as follows

(7) $${\hat{u}}_{i}\left(t\right)=K\left\{\Sigma_{i=1}^{N}l_{ij}\left[Gr_{j}\left(t\right)+G_{d}r_{j}\left(t-\tau_{1}\left(t\right)\right)\right]\right\},$$

where appropriate dimensions G, $G_{d}$ denote the coupling weights of the proper adjustments. The information on global network topology and wireless channel competition with propagation latency in the device-to-device path $\tau_{1}\left(t\right)$ is used to update the information sent to the centralized controllers. Then, the centralized controllers send the control instructions to the forwarding device at the source side based on analyzing the global network topology and wireless channel competition with a global view.

Therefore, all control instructions in the centralized controllers are expressed by Equation (7) to implement stable congestion control in the SDWN, which consists of topology information and the influence of the other forwarding devices with propagation latency in device-to-device paths.

Now, when the forwarding device at the source side receives the control instructions from the centralized controllers, the propagation latency in the device–controller pairs $\tau_{2}\left(t\right)$ must be considered. This means that the forwarding device makes an adjustment in latency $\tau_{2}\left(t\right)$ behind the centralized controllers sending the control instructions. Additionally, considering the presence of external disturbance, this closed-loop congestion control model can be converted into a robust control model. In addition, by combining Equations (4) with (6) as a data transmission process model and considering the global topology expressed by Equation (7), the linear closed-loop SDWN architecture incorporating the external disturbance can be described as follows

(8) $$\{\begin{array}{ll} {\dot{r}}_{i}\left(t\right)= & Ar_{i}\left(t\right)+A_{d}r_{i}\left(t-\tau_{1}\left(t\right)\right)+B_{u}u_{i}\left(t\right)+B_{w}w\left(t\right),\\ u_{i}\left(t\right)= & K\left\{r\left(t-\tau_{2}\left(t\right)\right)+\Sigma_{i=1}^{N}l_{ij}\left[Gr_{j}\left(t\right)+G_{d}r_{j}\left(t-\tau_{1}\left(t\right)\right)\right]\right\}, \end{array}$$

where A is the weight of the network parameters, and $B_{w}$ is the weight of the external disturbance. Let $Ar_{i}\left(t\right)=\hat{A}r_{i}\left(t\right)+\bar{A}r_{i}\left(t\right)$ and $u_{i}\left(t\right)={\hat{u}}_{i}\left(t\right)+\bar{u}\left(t\right)$.

Therefore, the congestion control system with two kinds of propagation latencies is converted into a robust congestion control model, which can be modeled by Equation (8).

### 3.4. A WOA-Based Scheduling Strategy Designed to Maximize Global SDWN Throughput

This section describes a WOA-based scheduling strategy designed to maximize global SDWN throughput, which can be non-preemptively pre-set to determine the network parameters of each forwarding device stabilization process.

#### 3.4.1. Whale Optimization Algorithm

First, the WOA algorithm, used as a preliminary strategy, is briefly introduced as follows. The WOA is a meta-heuristic optimization algorithm that simulates the foraging behavior of humpback whales, including their encircling of prey, bubble-net attacking strategies, and prey detection behavior [37,38,39,40].

##### Encircling Prey

Humpback whales have the capacity to identify the location of prey and hem them in. Owing to the optimal position designed such that it is not a priori, this paper assumes that the current best candidate solution is the location of prey. Each whale tries to update their position with respect to approaching to the prey. This foraging behavior can be modeled as follows.

$$\vec{D}=\vec{C}\cdot \left|\vec{X^{*}}\left(k\right)-\vec{X}\left(k\right)\right|,\;\vec{X}\left(k+1\right)=\vec{X^{*}}\left(k\right)-\vec{A}\cdot \vec{D},$$

where k indicates the kth iteration, $\vec{A}$ and $\vec{C}$ are coefficient vectors, the position vector of prey $X^{*}$ signifies the best solution, $\vec{X^{*}}$ is the position vector, |·| presents the absolute value, and · represents Hadamard’s product of vectors. In addition, $\vec{X^{*}}$ should be updated in each iteration if there is a better solution.

The vectors $\vec{A}$ and $\vec{C}$ are described as follows.

$$\vec{A}=2\vec{a}\cdot \vec{r},\;\vec{C}=2\vec{r},$$

where $\vec{r}\in \left[0,1\right]$ is a random vector, and $\vec{a}\in \left[0,2\right]$ is convergence vector from 2 to 0 with the iterations.

$$a=\left(2-2k/K_{max}\right),$$

where $K_{max}$ is the maximum number of the iterations.

##### Bubble-Net Attacking

**Figure 5:**
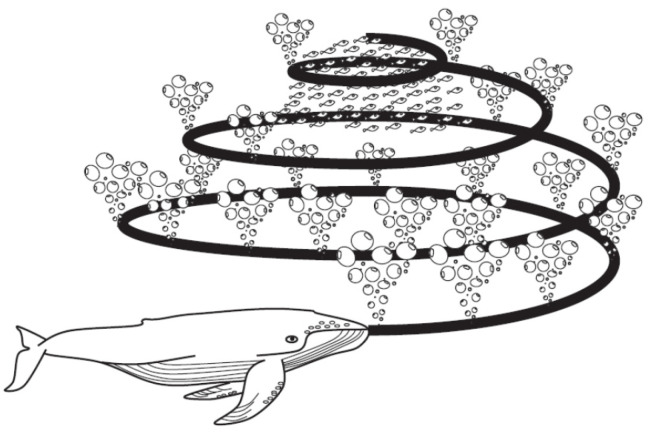
Bubble-net feeding behavior of humpback whales.

Figure 5 presents an image of the humpback whale’s hunting strategy, in which it prefers to attack its prey close to the surface. It swims down, generates bubbles in a spiral shape around its prey, and then dives up toward the surface to consume them.

While engaging in this foraging behavior, the whale generates distinctive bubbles arranged in a circle, including a coral loop, a lobtail, and a capture loop.

The WOA can be divided into two approaches:

1. Shrinking Encircling Mechanism

By analyzing the value of $\vec{a}$, our study can mathematically model the foraging behavior of a humpback whale.

2. Spiral Updating Position

By calculating the distance between the current position of the whale and the location of its prey, this approach mathematically simulates the whale’s foraging behavior, which can be expressed as follows.

$$\vec{X}\left(k+1\right)=\vec{D^{\prime }}\cdot e^{bl}\cdot \cos\left(2\pi l\right)+\vec{X^{*}}\left(k\right),$$

where $\vec{D}=\left|\vec{X^{*}}\left(k\right)-\vec{X}\left(k\right)\right|$ represents the distance of the ith whale to its prey; b, as a constant, indicates the shape of the logarithmic spiral; and l∈[0,1] is a random number. It has been reported that the whale swims around the prey within a shrinking circle and along a spiral-shaped path simultaneously.

Then, we assume that both the shrinking encircling mechanism and the spiral updating position can be selected with a probability of 50% to optimize the position of the whale.

Therefore, the bubble-net attacking method can be modelled as follows

$$\vec{X}\left(k+1\right)=\{\begin{array}{ll} \vec{X^{*}}\left(k\right)-\vec{A}\cdot \vec{D} & if\;p<0.5,\\ \vec{D^{\prime }}\cdot e^{bl}\cdot \cos\left(2\pi l\right)+\vec{X^{*}}\left(k\right) & if\;p\geq 0.5, \end{array}$$

where p∈[0,1] is a random value.

##### Search for Prey

As discussed in the above-mentioned analysis, the variation of the vector $\vec{A}$ is considered to be a critical parameter with respect to the random search for prey according to the position of each whale. Consequently, the whale should be forced to move far away from a reference position if $\left|\vec{A}\right|>1$. The position of the whale is updated according to a randomly selected whale rather than the best solution, for which a global search is performed. The model is described as follows.

$$\vec{D}=\vec{C}\cdot \left|{\vec{X}}_{rand}\left(k\right)-\vec{X}\left(k\right)\right|,\;\vec{X}\left(k+1\right)={\vec{X}}_{rand}\left(k\right)-\vec{A}\cdot \vec{D},$$

where ${\vec{X}}_{rand}\left(k\right)$ is a random position vector of a whale, which is selected from the current population.

#### 3.4.2. The Details of the WOA-Based Scheduling Strategy

The centralized controllers are considered to constitute a criterion device, in which a scheduling problem must be pre-set in order to solve the network congestion problem. Hence, each individual whale is considered to represent a specific scheduling plan in order to allocate appropriate sending rates at the source side based on the control instructions from the centralized controllers. After multiple iterations, the optimal individual whale output is selected as the best scheduling scheme according to an evaluation of the effectiveness of each scheduling scheme.

Ideally, the sending rate in each forwarding device remains stable and needs to be optimized under congestion control in order to maximize the global SDWN throughput with limited wireless network resources.

The specific steps of the WOA-based scheduling strategy are as follows.

Step 1. Represent the number of whales as N for defining all specific scheduling plans and consider a set of n forwarding devices $f_{1},f_{2},\cdots ,f_{n}$ that has a maximum processing capacity of $C_{i},i=1,2,\cdots ,n$. Suppose that only n levels of the sending rate exist at the source side with a weight $w_{i},i=1,2,\cdots ,n$.

Step 2. Configurate a set of m data flows $P_{j}\left(t\right),\;j=1,2,\cdots ,m$ waiting to be processed at the moment t. All of them must be processed and then transmitted to the destination.

Step 3. Suppose that $0<C_{1}\leq \cdots \leq C_{n}<+\infty$ and ignore the process latency of the forwarding devices to simplify the optimized scheduling model.

Step 4. Process each data flow $P_{j}$ using a series of forwarding devices (not all forwarding devices). Thus, define the processing data flow $P_{j}$ of these forwarding devices as $u_{ij}\in \left\{f_{1},\cdots ,f_{n}\right\}$, for which the maximum process capability is $C_{u_{ij}},i,j=1,2,\cdots ,n$, respectively.

Step 5. To maximize the global throughput, the optimized whale with an appropriate ideal rate of each forwarding device is denoted as $S_{i}\left(t\right),\;i=1,\;2,\;\cdots ,\;n$. Define $S\left(t\right)=\left\{S_{1}\left(t\right),S_{2}\left(t\right),\cdots ,S_{n}\left(t\right)\right\}$.

Therefore, the optimization problem can be described as follows.

(9) $$\begin{array}{c} \underset{w_{i}>0,S_{i}\left(t\right)>0}{\max}\sum_{i}S_{i}\left(t\right)\\ s.t.\;S_{i}=\sum_{j}C_{u_{ij}}\leq C_{i},\\ 0\leq C_{u_{ij}}\leq \min\left\{C_{i}\right\}=C_{1},\\ \frac{s_{1}\left(t\right)}{W_{1}}=\cdots =\frac{s_{i}\left(t\right)}{w_{i}}=\cdots =\frac{s_{k}\left(t\right)}{w_{k}}\\ \sum_{j}C_{u_{ij}}=P_{j}\left(t\right),\\ S_{i}\left(t\right),P_{j}\left(t\right),C_{i}\geq 0. \end{array}$$

The optimized whale with an appropriate ideal rate $S_{i}\left(t\right)$ of forwarding devices can be set under the above-mentioned constraints before data transmission in Equation (9). Initially, the optimized problem of maximizing the global SDWN throughput can be easily solved, and an uncomplicated allocated weighted proportion is defined as an ideal, optimized pre-set state by calculating each $S_{i}\left(t\right)$. Therefore, the WOA-based scheduling strategy has been presented to pre-set the goal of the stability congestion control scheme.

Based on the above-mentioned analysis, the stability congestion control model can be established. Then, external disturbance is incorporated to convert the stability congestion control model into a robust congestion control model in order to develop our global robust congestion control algorithm.

**Remark** **1.** *In this section, our study utilizes the WOA algorithm to implement the weighted fair scheduling strategy, which provides the control target of maximizing the global SDWN throughput for stability congestion control. The WOA algorithm can obtain solutions of required precision due to its low computational complexity and time consumption. Furthermore, a WOA algorithm that must be pre-set only provides the goal of the robust congestion control before executing the robust congestion control algorithm, which means that the WOA algorithm cannot be used in discussion regarding robust H∞ control performance.*

## 4. Problem Formulation of Robust Congestion Control

This section proposes a continuous, robust congestion control scheme with two kinds of propagation latencies in the SDWN. Its specific steps are as follows.

Step 1. The AIMD adjustment scheme is analyzed based on the congestion control system with propagation latency in device-to-device paths, and the values κ and ϑ are calculated after receiving the CS at the source side.

Step 2. A closed-loop congestion control model with propagation latency in device–controller pairs is proposed, and the data transmission process model is analyzed to provide decisions regarding control instructions in the centralized controllers.

Step 3. The effect of channel competition between neighboring forwarding devices is analyzed. Based on the global network topology and considering the effect of channel competition, all control instructions in the centralized controllers are expressed to stabilize congestion control.

Step 4. The WOA-based scheduling strategy is analyzed to calculate each optimized parameter value $S_{i}\left(t\right)$, and the target of the congestion control stability procedure is proposed in order to maximize the global SDWN throughput.

Step 5. Based on the first four steps, the stability congestion control model is established. Then, external disturbance is accounted for to convert the stability congestion control model into a robust congestion control model.

Step 6. A novel, robust H∞ control model is proposed to solve the robust congestion control model, thereby necessitating the determination of a robustness condition.

At the current stage, this paper focuses on establishing the robust H∞ control model and determining its robustness condition, which are introduced in the following two subsections.

### 4.1. Sending Rates at Source Side Approaching the Ideal Optimized Rates

Our study first considers the sending rates at the source side approaching the ideal optimized rates for establishing the robust H∞ control model. By means of optimized scheduling, the data flows are assigned for maximizing the throughput of global SDWN under the aforementioned satisfactory conditions. Then, the problem becomes keeping the optimization model network stable in the SDWN, which maintains an allocated weighted proportion at the source side according to the ideal optimized state S(t). If the global SDWN achieves robustness, it is stable at the maximal network throughput influenced by propagation latencies and external disturbance. Therefore, when S(t) is calculated and achieved, the sending rate of each forwarding device needs to approach its value.

It is preferred to keep the ideal optimized state S(t) stable under robust congestion control. The sending rate of each forwarding device needs to be unified as follows: $r_{1}\left(t\right)\to S_{1}\left(t\right),r_{2}\left(t\right)\to S_{2}\left(t\right),\cdots ,r_{n}\left(t\right)\to S_{n}\left(t\right)$($r_{i}\left(t\right)\to S_{i}\left(t\right),$ which means $\underset{t\to +\infty }{lim}\left|\left|r_{i}\left(t\right)\to S_{i}\left(t\right)\right|\right|=0$).

To unify the sending rates at the source side, the variable $x\left(t\right)={\left\{[r_{1}\left(t\right)-S_{1}\left(t\right){]}^{T},[r_{2}\left(t\right)-S_{2}\left(t\right)\right]}^{T},\cdots ,{\left[r_{n}\left(t\right)-S_{n}\left(t\right)\right]}^{T}\}$ is defined as the error state. Thus, the robust congestion control system (modeled by Equation (8)) can be converted into a global error system of the robust congestion control, which is described below:

$$\begin{array}{ll} {\dot{x}}_{i}\left(t\right)= & Ax_{i}\left(t\right)+A_{d}x_{i}\left(t-\tau_{1}\left(t\right)\right)+B_{u}K\{x\left(t-\tau_{2}\left(t\right)\right)\\ & +\Sigma_{i=1}^{N}l_{ij}\left[Gx_{j}\left(t\right)+G_{d}x_{j}\left(t-\tau_{1}\left(t\right)\right)\right]\}). \end{array}$$

### 4.2. Robust H∞ Control Model

Our study also considers the linear closed-loop SDWN with two kinds of propagation latency and external disturbance as the robust H∞ control model, which is described as follows.

(10) $$\{\begin{array}{ll} {\dot{x}}_{i}\left(t\right)= & Ax_{i}\left(t\right)+A_{d}x_{i}\left(t-\tau_{1}\left(t\right)\right)+B_{u}u_{i}\left(t\right)+B_{w}w\left(t\right)\\ u_{i}\left(t\right)= & K\left\{x\left(t-\tau_{2}\left(t\right)\right)+\Sigma_{i=1}^{N}l_{ij}\left[Gx_{j}\left(t\right)+G_{d}x_{j}\left(t-\tau_{1}\left(t\right)\right)\right]\right\}\\ z\left(t\right)= & Ix\left(t\right)\\ x\left(t\right)= & \phi \left(t\right), \end{array}$$

where $x_{i}\left(t\right)\in {\mathbb{R}}^{n}$ is the error state denoting the state of the difference between the real-time state and the ideal optimized pre-set state; $u_{i}\left(t\right)\in {\mathbb{R}}^{n}$ represents the control instruction in the centralized controllers; z(t) is the controlled output, which can reflect the energy trajectory; w(t) is the external disturbance with a covariance matrix equal to w and expectation equal to zero; and $\tau_{i}\left(t\right),i=1,2$ as the continuous time satisfies

$$0\leq \tau_{i}\leq h_{i},\;\mu_{mi},\leq \dot{\tau }\left(t\right)\leq \mu_{Mi},$$

where $\mu_{mi},\mu_{Mi}$, and $h_{i},i=1,2$ are constants.

At this stage, definitions required for the analysis of the robust H∞ control model are provided as follows.

**Definition** **2.** *There exists a description of the energy relation between the controlled output z(t) and the external disturbance output w(t). By considering the real SDWN, the energy relationship of these two outputs is believed to be $\int_{0}^{\infty }\{z^{T}(t)z\left(t\right)-\gamma^{2}w^{T}\left(t\right)w\left(t\right)\}dt,\;||T_{wz}\left(z\right){||}_{\infty }<\gamma$, where γ is a prescribed positive scalar. This shows that the energy of the external disturbance has been absorbed after being controlled, which implies that robust H∞ control has been achieved.*

**Lemma** **1** (Kronecker product): *Let ⊗ denote the notation of Kronecker product. Accordingly, the following properties are satisfied in appropriate dimensions:*

*(i)* 
(αA)⊗B=A⊗(αB),

*(ii)* 
(A+B)⊗C=A⊗C+B⊗C,

*(iii)* 
(A⊗B)(C⊗D)=(AC)⊗(BD).

**Lemma** **2** [51]: *For any matrix R>0 and a vector function $x:\left[\alpha ,\beta \right]\to {\mathbb{R}}^{n}$, if the integrals concerned are well defined, the following inequality holds:*

$$\{\left[\int_{\alpha }^{\beta }x^{T}\left(s\right)ds\right]R\left[\int_{\alpha }^{\beta }x\left(s\right)ds\right]\leq \left(\beta -\alpha \right)\int_{\alpha }^{\beta }x^{T}\left(s\right)Rx\left(s\right)ds.$$

**Lemma** **3** [52]: *For any matrices $R\in {\mathbb{R}}^{n\times n},\;Y\in {\mathbb{R}}^{2n\times n}$, and $X\in {\mathbb{R}}^{2n\times 2n}$ with $\left[\begin{array}{ll} X & Y\\ {}* & R \end{array}\right]\geq 0$, and a vector function $x:\left[\alpha ,\beta \right]\to {\mathbb{R}}^{n}$, if the integrals concerned are well defined, then the following inequality holds:*

$$-\int_{\alpha }^{\beta }{\dot{x}}^{T}\left(s\right)R\dot{x}\left(s\right)\leq \vartheta^{T}\left[He\left\{Y\Pi \right\}+\left(\beta -\alpha \right)X\right]\vartheta ,$$

**Lemma** **4** [53]: *For any matrix R>0 and a differentiable signal x in $\left[\alpha ,\beta \right]\to {\mathbb{R}}^{n}$, the following inequality holds:*

$$-\int_{\alpha }^{\beta }{\dot{x}}^{T}\left(s\right)Rx\left(s\right)ds\leq \varpi^{T}\hat{\Omega }\varpi /\left(\beta -\alpha \right),$$

*where*

$$\hat{\Omega }=\left[\begin{array}{ccc} -4R & -2R & 6R\\ {}* & -4R & 6R\\ {}* & * & -12R \end{array}\right],$$

$$\varpi =\left[x^{T}\left(\beta \right)\;x^{T}\left(\alpha \right)\;\int_{\alpha }^{\beta }x^{T}\left(s\right)ds/\left(\beta -\alpha \right)\right].$$

**Lemma** **5** [54]: *Let x be a differentiable function: $\left[\alpha ,\beta \right]\to {\mathbb{R}}^{n}$. For symmetric matrices $R\in {\mathbb{R}}^{n\times n}$ and $Z_{1},\;Z_{3}\in {\mathbb{R}}^{3n\times 3n}$, and any matrices $Z_{2}\in {\mathbb{R}}^{3n\times 3n}$ and $N_{1},\;N_{2}\in {\mathbb{R}}^{3n\times n}$ satisfying*

$$\bar{\Psi }=\left[\begin{array}{ccc} Z_{1} & Z_{2} & N_{1}\\ {}* & Z_{3} & N_{2}\\ {}* & * & R \end{array}\right]\geq 0,$$

*the following inequality holds:*

$$-\int_{\alpha }^{\beta }{\dot{s}}^{T}\left(t\right)R\dot{x}\left(s\right)ds\leq \varpi^{T}\bar{\Omega }\varpi ,$$

*where*

$$\begin{array}{l} \bar{\Omega }=\left(\beta -\alpha \right)\left(Z_{1}+Z_{3}/3\right)+He\left\{N_{1}\bar{\Pi_{1}}+N_{2}\bar{\Pi_{2}}\right\},\\ {\bar{\Pi }}_{1}={\bar{e}}_{1}-{\bar{e}}_{2},\;{\bar{\Pi }}_{2}=2{\bar{e}}_{3}-{\bar{e}}_{1}-{\bar{e}}_{2},\\ {\bar{e}}_{1}=\left[I\;0\;0\right],\;{\bar{e}}_{2}=\left[0\;I\;0\right],\;{\bar{e}}_{3}=\left[0\;0\;I\right], \end{array}$$

*and ϖ is similarly defined in **Lemma 4**.*

## 5. Criterion and Robustness Condition of Robust H∞ Control Model in the SDWN

In this section, a new criterion of a robust H∞ control model in the SDWN is proposed to determine a robustness condition using matrix-based integral inequality. The criterion is first established based on the LMI control toolbox. We provide the following definition:

$$\begin{array}{l} \tilde{A}=I_{N}⊗A,\;{\tilde{A}}_{d}=I_{N}⊗A_{d},\;\tilde{B}=I_{N}⊗B_{w},\;{\tilde{B}}_{u}=I_{N}⊗B_{u},\\ \tilde{G}=G⊗L,\;{\tilde{G}}_{d}=G_{d}⊗L. \end{array}$$

According to the Kronecker product, the second-order system can be rewritten as a new, robust H∞ control model.

(11) $$\{\begin{array}{ll} \dot{x}\left(t\right)= & \tilde{A}x\left(t\right)+{\tilde{A}}_{d}x\left(t-\tau_{1}\left(t\right)\right)+u\left(t\right)+\tilde{B}w\left(t\right)\\ u\left(t\right)= & K\left\{{\tilde{B}}_{u}x\left(t-\tau_{2}\left(t\right)\right)+\tilde{G}x\left(t\right)+{\tilde{G}}_{d}x\left(t-\tau_{1}\left(t\right)\right)\right\}\\ z\left(t\right)= & I_{N}x\left(t\right)\\ x\left(t\right)= & \phi \left(t\right). \end{array}$$

The following nomenclature for vectors and matrices simplifies the representation:

$$\begin{array}{lll} \eta_{1}\left(t\right) & = & {\left[\eta_{3}^{T}\int_{t-\tau_{1}\left(t\right)}^{t}x^{T}\left(s\right)ds\int_{t-h_{1}}^{t-\tau_{1}\left(t\right)}x^{T}\left(s\right)ds\right]}^{T},\\ \eta_{2}\left(t\right) & = & {\left[x^{T}\left(t\right){\dot{x}}^{T}\left(t\right)\right]}^{T},\\ \eta_{3}\left(t\right) & = & {\left[x^{T}\left(t\right)x^{T}\left(t-\tau_{1}\left(t\right)\right)\;x^{T}\left(t-h_{1}\right)\;x^{T}\left(t-\tau_{2}\left(t\right)\right)\;x^{T}\left(t-h_{2}\right)\right]}^{T},\\ \eta_{4}\left(t\right) & = & {\left[{\dot{x}}^{T}\left(t\right)\;{\dot{x}}^{T}\left(t-\tau_{1}\left(t\right)\right)\;{\dot{x}}^{T}\left(t-h_{1}\right)\;{\dot{x}}^{T}\left(t-\tau_{2}\left(t\right)\right)\;{\dot{x}}^{T}\left(t-h_{2}\right)\right]}^{T},\\ \eta_{5}\left(t\right) & = & {\left[\int_{t-\tau_{1}\left(t\right)}^{t}x^{T}\left(s\right)ds/\tau_{1}\left(t\right)\;\int_{t-h_{1}}^{t-\tau_{1}\left(t\right)}x^{T}\left(s\right)ds/\left(h_{1}-\tau_{1}\left(t\right)\right)\right]}^{T},\\ \eta_{6}\left(t\right) & = & {\left[\int_{t-\tau_{2}\left(t\right)}^{t}x^{T}\left(s\right)ds/\tau_{2}\left(t\right)\;\int_{t-h_{2}}^{t-\tau_{2}\left(t\right)}x^{T}\left(s\right)ds/\left(h_{2}-\tau_{2}\left(t\right)\right)\right]}^{T},\\ \xi \left(t\right) & = & {\left[\eta_{3}^{T}\left(t\right)\;\eta_{4}^{T}\left(t\right)\;\eta_{5}^{T}\left(t\right)\;\eta_{6}^{T}\left(t\right)\right]}^{T},\\ \xi \left(t\right) & = & {\left[{\bar{\xi }}^{T}\left(t\right)\;w^{T}\left(t\right)\right]}^{T},\\ e_{i} & = & \left[0_{n\times \left(i-1\right)n}\;I_{n}\;0_{n\times \left(15-i\right)n}\right],\;i=1,2,\cdots ,15. \end{array}$$

**Theorem** **1.** *For given scalars $h_{1},h_{2}>0$ and $\mu_{11}<{\dot{d}}_{1}\left(t\right)<\mu_{12}<1,\mu_{21}<{\dot{d}}_{2}\left(t\right)<\mu_{22}<1,$ the global error system is robust under robust congestion control if there exist appropriate dimension matrices $P>0,\;Q_{i}\geq 0$ and R>0,i=1,2, appropriate dimension symmetrical matrices $X_{11}^{\left(i\right)},X_{13}^{\left(i\right)},X_{21}^{\left(i\right)},X_{23}^{\left(i\right)},i=1,2,$ and any matrices $X_{12}^{\left(i\right)},X_{22}^{\left(i\right)},N_{11}^{\left(i\right)},N_{12}^{\left(i\right)},N_{21}^{\left(i\right)},N_{21}^{\left(i\right)},i=1,2$, thus allowing the following conditions in (12) to (13) to hold:*

(12) Ξ<0,

(13) $$\Psi_{1}^{\left(1\right)}=\left[\begin{array}{ccc} X_{11}^{\left(1\right)} & X_{12}^{\left(1\right)} & N_{11}^{\left(1\right)}\\ {}* & X_{13}^{\left(1\right)} & N_{12}^{\left(1\right)}\\ {}* & * & R_{1} \end{array}\right]\geq 0,\Psi_{2}^{\left(1\right)}=\left[\begin{array}{ccc} X_{21}^{\left(1\right)} & X_{22}^{\left(1\right)} & N_{21}^{\left(1\right)}\\ {}* & X_{23}^{\left(1\right)} & N_{22}^{\left(1\right)}\\ {}* & * & R_{1} \end{array}\right]\geq 0,\;\Psi_{1}^{\left(2\right)}=\left[\begin{array}{ccc} X_{11}^{\left(2\right)} & X_{12}^{\left(2\right)} & N_{11}^{\left(2\right)}\\ {}* & X_{13}^{\left(2\right)} & N_{12}^{\left(2\right)}\\ {}* & * & R_{2} \end{array}\right]\geq 0,\Psi_{2}^{\left(2\right)}=\left[\begin{array}{ccc} X_{21}^{\left(2\right)} & X_{22}^{\left(2\right)} & N_{21}^{\left(2\right)}\\ {}* & X_{23}^{\left(2\right)} & N_{22}^{\left(2\right)}\\ {}* & * & R_{2} \end{array}\right]\geq 0,$$

*where*

$$\begin{array}{ll} \Xi = & He\{\Pi_{1}^{T}P\Pi_{18}+\Pi_{6}^{T}N_{11}^{\left(1\right)}\Pi_{10}+\Pi_{6}^{T}N_{12}^{\left(1\right)}\Pi_{11}+\Pi_{7}^{T}N_{21}^{\left(1\right)}\Pi_{12}+\Pi_{7}^{T}N_{22}^{\left(1\right)}\Pi_{13}+\Pi_{8}^{T}N_{11}^{\left(2\right)}\Pi_{14}\\ & +\Pi_{8}^{T}N_{12}^{\left(2\right)}\Pi_{15}+\Pi_{9}^{T}N_{21}^{\left(2\right)}\Pi_{16}+\Pi_{9}^{T}N_{12}^{\left(2\right)}\Pi_{17}\}-\left(1-\dot{\tau }\left(t\right)\right)\Pi_{4}^{T}\left(Q_{1}-Q_{2}\right)\Pi_{4}-\Pi_{5}^{T}Q_{2}\Pi_{5}\\ & +\overset{2}{\underset{j=1}{\sum }}h_{j}e_{6}^{T}R_{j}e_{6}-\gamma^{2}e_{15e_{15}}^{T}+\tau_{1}\left(t\right)\Pi_{6}^{T}\left(X_{11}^{\left(1\right)}+\frac{X_{13}^{\left(1\right)}}{3}\right)\Pi_{6}\\ & +\left(h_{1}-\tau_{1}\left(t\right)\right)\Pi_{7}^{T}\left(X_{21}^{\left(1\right)}+\frac{X_{23}^{\left(1\right)}}{3}\right)\Pi_{7}+\tau_{2}\left(t\right)\Pi_{8}^{T}\left(X_{11}^{\left(2\right)}+\frac{X_{13}^{\left(2\right)}}{3}\right)\Pi_{8}\\ & +\left(h_{1}-\tau_{1}\left(t\right)\right)\Pi_{9}^{T}\left(X_{21}^{\left(2\right)}+\frac{X_{23}^{\left(2\right)}}{3}\right)\Pi_{9}+\Pi_{1}^{T}P\Pi_{19}+\Pi_{1}^{T}P\Pi_{20}\\ & +\Pi_{1}^{T}P\Pi_{21}+\Pi_{3}^{T}Q_{1}\Pi_{3}+\Pi_{22}^{T}\Pi_{22}+e_{1}^{T}e_{1} \end{array}$$

*and*

$$\begin{array}{ll} \Pi_{1}= & {\left[e_{1}^{T}\;e_{2}^{T}\;e_{3}^{T}\;e_{4}^{T}\;e_{5}^{T}\;\tau_{1}\left(t\right)e_{11}^{T}\;\left(h-\tau_{1}\left(t\right)\right)e_{12}^{T}\right]}^{T},\\ \Pi_{2}= & {\left[e_{6}^{T}\;e_{7}^{T}\;e_{8}^{T}\;e_{9}^{T}\;e_{10}^{T}\;e_{1}^{T}-\left(1-\dot{\tau }\left(t\right)\right)e_{2}^{T}\;\left(1-\dot{\tau }\left(t\right)\right)e_{2}^{T}-e_{3}^{T}\right]}^{T},\\ \Pi_{3}= & {\left[e_{1}^{T}\;e_{4}^{T}\right]}^{T},\\ \Pi_{4}= & {\left[e_{2}^{T}\;e_{5}^{T}\right]}^{T},\\ \Pi_{5}= & {\left[e_{3}^{T}\;e_{6}^{T}\right]}^{T},\\ \Pi_{6}= & {\left[e_{1}^{T}\;e_{2}^{T}\;e_{11}^{T}\right]}^{T},\\ \Pi_{7}= & {\left[e_{2}^{T}\;e_{3}^{T}\;e_{12}^{T}\right]}^{T},\\ \Pi_{8}= & {\left[e_{1}^{T}\;e_{4}^{T}\;e_{13}^{T}\right]}^{T},\\ \Pi_{9}= & {\left[e_{4}^{T}\;e_{5}^{T}\;e_{14}^{T}\right]}^{T},\\ \Pi_{10}= & e_{1}-e_{2},\\ \Pi_{11}= & 2e_{11}-e_{1}-e_{2},\\ \Pi_{12}= & e_{2}-e_{3},\\ \Pi_{13}= & 2e_{12}-e_{2}-e_{3},\\ \Pi_{14}= & e_{1}-e_{4},\\ \Pi_{15}= & 2e_{13}-e_{1}-e_{4},\\ \Pi_{16}= & e_{4}-e_{5},\\ \Pi_{17}= & 2e_{14}-e_{4}-e_{5},\\ \Pi_{18}= & {\left[\left(\tilde{A}+\tilde{G}\right)e_{1}^{T}\;e_{7}^{T}\;e_{8}^{T}\;e_{9}^{T}\;e_{10}^{T}\;e_{1}^{T}-\left(1-\dot{\tau }\left(t\right)\right)e_{2}^{T}\;\left(1-\dot{\tau }\left(t\right)\right)e_{2}^{T}-e_{3}^{T}\right]}^{T},\\ \Pi_{19}= & [\left({\tilde{A}}_{d}+{\tilde{G}}_{d}\right)e_{2}^{T}\;e_{7}^{T}\;e_{8}^{T}\;e_{9}^{T}\;e_{10}^{T}\;e_{1}^{T}-\left(1-\dot{\tau }\left(t\right)\right)e_{2}^{T}\;\left(1-\dot{\tau }\left(t\right)\right)e_{2}^{T}\\ \Pi_{20}= & {\left[K{\tilde{B}}_{u}e_{4}^{T}\;e_{7}^{T}\;e_{8}^{T}\;e_{9}^{T}\;e_{10}^{T}\;e_{1}^{T}-\left(1-\dot{\tau }\left(t\right)\right)e_{2}^{T}\;\left(1-\dot{\tau }\left(t\right)\right)e_{2}^{T}-e_{3}^{T}\right]}^{T},\\ \Pi_{21}= & {\left[\tilde{B}e_{15}^{T}\;e_{7}^{T}\;e_{8}^{T}\;e_{9}^{T}\;e_{10}^{T}\;e_{1}^{T}-\left(1-\dot{\tau }\left(t\right)\right)e_{2}^{T}\;\left(1-\dot{\tau }\left(t\right)\right)e_{2}^{T}-e_{3}^{T}\right]}^{T},\\ \Pi_{22}= & {\left[e_{1}^{T}\;e_{15}^{T}\right]}^{T}. \end{array}$$

**Proof:**  Consider the following Lyapunov–Krasovskii functionals acting on this closed-loop robust H∞ control model:

$$\begin{array}{ll} V\left(t\right)= & \eta_{1}^{T}\left(t\right)P\eta_{1}\left(t\right)+\int_{t-\tau_{1}\left(t\right)}^{t}x^{T}\left(s\right)Q_{1}x\left(s\right)ds+\int_{t-h_{1}}^{t-\tau_{1}\left(t\right)}x^{T}\left(s\right)Q_{2}x\left(s\right)ds\\ & +\overset{2}{\underset{j=1}{\sum }}\int_{-h_{j}}^{0}\int_{t+\theta }^{t}{\dot{x}}^{T}\left(s\right)R_{j}\dot{x}\left(s\right)dsd\theta . \end{array}$$

The derivative of V(t) is

$$\begin{array}{cc} \dot{V}\left(t\right)= & {\bar{\xi }}^{T}\left(t\right)\{He\Pi_{1}P\Pi_{2}+\Pi_{3}^{T}Q_{1}\Pi_{3}-\left(1-\dot{\tau }\left(t\right)\right)\Pi_{4}^{T}\left(Q_{1}-Q_{2}\right)\Pi_{4}\\ & -\Pi_{5}^{T}Q_{2}\Pi_{5}+\overset{2}{\underset{j=1}{\sum }}h_{j}e_{6}^{T}R_{j}e_{6}\}\bar{\xi }\left(t\right)-\overset{2}{\underset{j=1}{\sum }}\int_{t-h_{j}}^{t}{\dot{x}}^{T}\left(s\right)R_{j}\dot{x}\left(s\right)ds\\ = & {\bar{\xi }}^{T}\left(t\right)\Xi_{1}\bar{\xi }\left(t\right)-\overset{2}{\underset{j=1}{\sum }}\int_{t-h_{j}}^{t}{\dot{x}}^{T}\left(s\right)R_{j}\dot{x}\left(s\right)ds. \end{array}$$

Since $\Psi_{j}^{\left(i\right)}\geq 0,\;i,j=1,2$ in Lemma 5, the following is yielded:

$$\begin{array}{ll} & -\overset{2}{\underset{j=1}{\sum }}\int_{t-h_{j}}^{t}{\dot{x}}^{T}\left(s\right)R_{j}\dot{x}\left(s\right)ds\\ = & -\int_{t-\tau_{1}\left(t\right)}^{t}{\dot{x}}^{T}\left(s\right)R_{j}\dot{x}\left(s\right)ds-\int_{t-h_{1}}^{t-\tau_{1}\left(t\right)}{\dot{x}}^{T}\left(s\right)R_{j}\dot{x}\left(s\right)ds\\ & -\int_{t-\tau_{2}\left(t\right)}^{t}{\dot{x}}^{T}\left(s\right)R_{j}\dot{x}\left(s\right)ds-\int_{t-h_{2}}^{t-\tau_{2}\left(t\right)}{\dot{x}}^{T}\left(s\right)R_{j}\dot{x}\left(s\right)ds\\ \leq & {\bar{\xi }}^{T}\left(t\right)\{\tau_{1}\left(t\right)\Pi_{6}^{T}\left(X_{11}^{\left(1\right)}+X_{13}^{\left(1\right)}/3\right)\Pi_{6}+\left(h_{1}-\tau_{1}\left(t\right)\right)\Pi_{7}^{T}\left(X_{21}^{\left(1\right)}+X_{23}^{\left(1\right)}/3\right)\Pi_{7}\\ & +\tau_{2}\left(t\right)\Pi_{8}^{T}\left(X_{11}^{\left(2\right)}+X_{13}^{\left(2\right)}/3\right)\Pi_{8}+\left(h_{1}-\tau_{1}\left(t\right)\right)\Pi_{9}^{T}\left(X_{21}^{\left(2\right)}+X_{23}^{\left(2\right)}/3\right)\Pi_{9}\\ & +He\{\Pi_{6}^{T}N_{11}^{\left(1\right)}\Pi_{10}+\Pi_{6}^{T}N_{12}^{\left(1\right)}\Pi_{11}+\Pi_{7}^{T}N_{21}^{\left(1\right)}\Pi_{12}+\Pi_{7}^{T}N_{22}^{\left(1\right)}\Pi_{13}\\ & +\Pi_{8}^{T}N_{11}^{\left(2\right)}\Pi_{14}+\Pi_{8}^{T}N_{12}^{\left(2\right)}\Pi_{15}+\Pi_{9}^{T}N_{21}^{\left(2\right)}\Pi_{16}+\Pi_{9}^{T}N_{12}^{\left(2\right)}\Pi_{17}\}\}\bar{\xi }\left(t\right) \end{array}$$

Thus, the continuous linear closed-loop congestion control system (10) is considered, and the derivative of V(t) is

$$\dot{V}\left(t\right)=\xi \left(t\right)\bar{\Xi }\xi \left(t\right)+\gamma^{2}w^{T}\left(t\right)w\left(t\right)=\xi^{T}\left(t\right)\left\{\bar{\Xi }+\Pi_{22}^{T}\Pi_{22}\right\}\xi \left(t\right),$$

where

$$\begin{array}{ll} \bar{\Xi }= & He\{\Pi_{1}^{T}P\Pi_{18}+\Pi_{6}^{T}N_{11}^{\left(1\right)}\Pi_{10}+\Pi_{6}^{T}N_{12}^{\left(1\right)}\Pi_{11}+\Pi_{7}^{T}N_{21}^{\left(1\right)}\Pi_{12}+\Pi_{7}^{T}N_{22}^{\left(1\right)}\Pi_{13}\\ & +\Pi_{8}^{T}N_{11}^{\left(2\right)}\Pi_{14}+\Pi_{8}^{T}N_{12}^{\left(2\right)}\Pi_{15}+\Pi_{9}^{T}N_{21}^{\left(2\right)}\Pi_{16}+\Pi_{9}^{T}N_{12}^{\left(2\right)}\Pi_{17}\}\\ & +\Pi_{1}^{T}P\Pi_{19}+\Pi_{1}^{T}P\Pi_{20}+\Pi_{1}^{T}P\Pi_{21}+\Pi_{3}^{T}Q_{1}\Pi_{3}-\left(1-\dot{\tau }\left(t\right)\right)\Pi_{4}^{T}\left(Q_{1}-Q_{2}\right)\Pi_{4}\\ & -\Pi_{5}^{T}Q_{2}\Pi_{5}+\overset{2}{\underset{j=1}{\sum }}h_{j}e_{6}^{T}R_{j}e_{6}-\gamma^{2}e_{15e_{15}}^{T}+\tau_{1}\left(t\right)\Pi_{6}^{T}\left(X_{11}^{\left(1\right)}+X_{13}^{\left(1\right)}/3\right)\Pi_{6}\\ & +\left(h_{1}-\tau_{1}\left(t\right)\right)\Pi_{7}^{T}\left(X_{21}^{\left(1\right)}+X_{23}^{\left(1\right)}/3\right)\Pi_{7}+\tau_{2}\left(t\right)\Pi_{8}^{T}\left(X_{11}^{\left(2\right)}+X_{13}^{\left(2\right)}/3\right)\Pi_{8}\\ & +\left(h_{1}-\tau_{1}\left(t\right)\right)\Pi_{9}^{T}\left(X_{21}^{\left(2\right)}+X_{23}^{\left(2\right)}/3\right)\Pi_{9}. \end{array}$$

The following H∞ performance index J is considered:

$$J=\int_{0}^{\infty }\{z^{T}(t)z\left(t\right)-\gamma^{2}w^{T}\left(t\right)w\left(t\right)\}dt,||T_{wz}\left(z\right){||}_{\infty }<\gamma ,$$

where γ is a prescribed positive scalar.

H∞ performance index J displays the energy relationship between the controlled output and the external interference. J<0 indicates that the energy of external interference has been expended under the control law and that robust control has been achieved.

Consider w(t)≠0, where the following relation bas been obtained:

$$\dot{V}\left(t\right)+z^{T}\left(t\right)z\left(t\right)-\gamma^{2}w^{T}\left(t\right)w\left(t\right)\leq \xi^{T}\left(t\right)\left\{\bar{\Xi }+\Pi_{22}^{T}\Pi_{22}+e_{1}^{T}e_{1}\right\}\xi \left(t\right)=\xi^{T}\left(t\right)\Xi \xi \left(t\right).$$

Following from inequalities (12) and (13) and the Schur Complement Lemma, we obtain

$$\dot{V}\left(t\right)+z^{T}\left(t\right)z\left(t\right)-\gamma^{2}w^{T}\left(t\right)w\left(t\right)<0.$$

Sum t from 0 to ∞ so that

$$\int_{0}^{\infty }\{z^{T}(t)z\left(t\right)-\gamma^{2}w^{T}\left(t\right)w\left(t\right)\}dt<V\left(0\right)-V\left(\infty \right).$$

With the zero initial condition V(0)=0, we obtain

$$\int_{0}^{\infty }\{z^{T}(t)z\left(t\right)-\gamma^{2}w^{T}\left(t\right)w\left(t\right)\}dt<0.$$

Based on the Lyapunov–Krasovskii theory, the continuous linear delay closed-loop system with external interference can achieve robust control J<0 with a desirable H∞ performance index $|\left|T_{wz}\left(z\right)\right|{|}_{\infty }<\gamma$ according to (12) and (13).

Thus, the proof is complete. □

## 6. Simulation

In Section 6, a numerical network simulation is conducted to illustrate the effectiveness of our proposed robust congestion control scheme in the SDWN scenario and the robustness conditions given in Theorem 1. Furthermore, a comparison with the AIMD adjustment scheme and the scheme incorporating information-forwarding and the control algorithm is given to demonstrate the superiority of our proposed robust congestion control scheme over the other congestion control approaches that are applied in SDWNs to maximize global SDWN throughput and thus maintain long-term stability with two kinds of propagation latency and external disturbance.

### 6.1. Scenario Establishment

**Figure 6:**
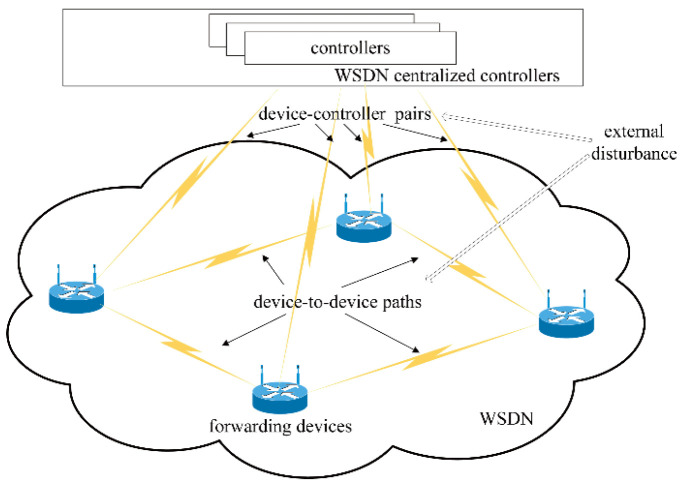
A typical SDWN scenario for the numerical simulation.

An SDWN scenario is established for the numerical simulation (as shown in Figure 6), which consists of a group of SDWN-centralized controllers and four forwarding devices. Each forwarding device is connected to one another and the centralized controllers by a wireless channel. In the wireless channel, the influence of propagation latency and external disturbance is present in every device-to-device path and device–controller pair. Thus, the forwarding devices first record their CS information and periodically broadcast it to the centralized controllers. Next, the centralized controllers yield control policies for robust congestion control and send control instructions to the four forwarding devices. Finally, the four forwarding devices follow these control instructions and make proper adjustments to the sending rates at the source side in order to achieve the desired global robust control for network congestion in the SDWN.

### 6.2. Parameter Description

Now, to analyze Theorem 1 (inequations (12) and (13)), the parameters in Equation (11) are considered as follows.

$$k=1.55,\;\vartheta =16.5,A=\left[\begin{array}{cc} 0 & 1\\ 0 & -k \end{array}\right],\;A_{d}=\left[\begin{array}{cc} 0 & 0\\ -\vartheta & 0 \end{array}\right],$$

and $B_{w}={\left(2.2,1.4,3.6,2.8,1.2,2.4,0.6,1.8\right)}^{T}$. The inner coupling matrices are defined as $G=diag\left\{3,2\right\},G_{d}=diag\left\{3,5\right\}$, and the coupling matrix L is as follows

$$k=1.55,\;\vartheta =16.5,A=\left[\begin{array}{cc} 0 & 1\\ 0 & -k \end{array}\right],\;A_{d}=\left[\begin{array}{cc} 0 & 0\\ -\vartheta & 0 \end{array}\right],$$

and $B_{w}={\left(2.2,1.4,3.6,2.8,1.2,2.4,0.6,1.8\right)}^{T}$. Define the inner coupling matrices as $G=diag\left\{3,2\right\},G_{d}=diag\left\{3,5\right\},$ and the coupling matrix L is as follows.

$$L=\left[\begin{array}{cccc} -3 & 1 & 1 & 1\\ 1 & -2 & 1 & 0\\ 1 & 1 & -3 & 1\\ 1 & 0 & 1 & -2 \end{array}\right].$$

A robust congestion control scheme in SDWN is researched in this paper. In addition, recall that an essential precondition for solving the re-stabilization problem is determined by global asymptotic stability. As a necessary condition for global asymptotic stability, x(t) indicates that the error state satisfies $\underset{t\to 0}{lim}\;x\left(t\right)=0$. Therefore, we consider a stability congestion control pattern with zero initial conditions to correspond to $x\left(t\right)={\left(0\;0\;0\;0\;0\;0\;0\;0\right)}^{T}$.

A four-dimensional matrix is proposed to describe four forwarding devices under robust H∞ control in the SDWN. There exists a feasible solution to LMIs (inequations (12) and (13)) according to Theorem 1. Now, suppose the control strength K=diag{2.5,1.2,1.8,1.6,2,2.5,2.2,5} and the control instruction $B_{u}=\left[\begin{array}{cc} 1 & 2\\ 0.3 & 2 \end{array}\right]$.

To render the simulation tractable, the external disturbance output is denoted as follows:

$$w\left(t\right)=\{\begin{array}{ll} 1 & 0\leq t\leq 1,\\ 0 & otherwise, \end{array}$$

which is a function with limited energy and duration.

### 6.3. Simulation Results

We conducted experiments on both the variations in the error states and the energy trajectories in the robust H∞ control scheme in the SDWN. The experiments included the AIMD adjustment scheme and the information-forwarding and control algorithm as the benchmark algorithms.

Now, short descriptions of all the benchmark algorithms under the condition of **Theorem 1** are given as follows.

**Short descriptions of all the benchmark algorithms**

- **AIMD adjustment scheme** [28,29]: This is a traditional network control scheme, which is often used at the source side without considering both the propagation latency in device–controller pairs and channel competition in order to achieve network robustness with a global view of the SDWN.
- **Information forwarding and control algorithm** [36,37,38]: The previous algorithm that addresses network congestion implements the robust control of partial networks instead of control incorporating a global view of the SDWN, which is often used for robust congestion control without considering the propagation latency in device–controller pairs.

The definitions of both the variation in the error state and energy trajectory are given as follows.

**Definition** **3.** ***Variation in error state:** The variation in error state is defined as the trajectory of the current state approaching the ideal state in SDWNs, which is believed to be close to 0 when the second-order system is under robust congestion control. Moreover, due to the characteristics of the second-order system, each variation in the error state is described by two trajectories that should converge at 0, i.e., the error equals 0 over a period of time.*

**Definition** **4.** ***Energy trajectory:** Energy trajectory is defined as the trajectory of the energy output, which is utilized to describe the energy trajectories of the controlled output z(t) and the external disturbance w(t). As J<0, a necessary condition for the achievement of robust H∞ congestion control is that the energy of the external disturbance must not have been absorbed after being controlled, and its corollary is that the controlled output z(t) is lower than the external disturbance output w(t) after being controlled.*

**Figure 7:**
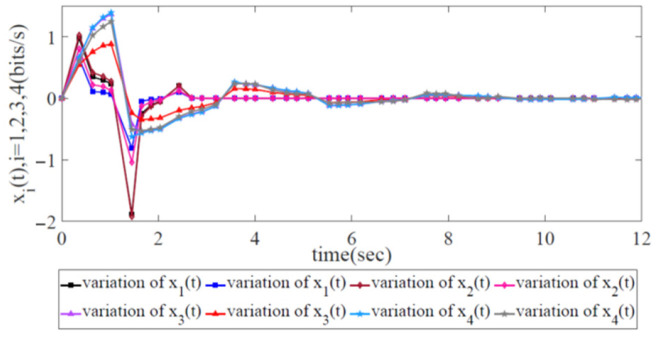
Variation in error state $x_{i}\left(t\right),i=1,2,3,4$ with two trajectories to represent its variation with two kinds of propagation latencies and external disturbance.

According to Theorem 1, there exists a feasible solution to LMIs from Inequality (12) to Inequality (13). The variation in the error state $x_{i}\left(t\right),i=1,2,3,4$ with two trajectories is displayed in Figure 7, each of which, as second-order systems, has two trajectories to display its variations. Notably, all the sending rates at the source side are initially stable, and the value of all the error states is initially zero due to the global asymptotic stability of the SDWN. Next, when the external disturbance appears at the initial moment, jitter occurs, and all error states become unstable. This indicates that all the source forwarding devices adopted the robust congestion control scheme to carry out the sending rate adjustments for re-stabilization under the presence of two kinds of propagation latency and external disturbance. Then, the error states converge after a finite length of time. This indicates that the error state x(t) is under robust congestion control and that the whole wireless network has achieved global SDWN robustness.

**Figure 8:**
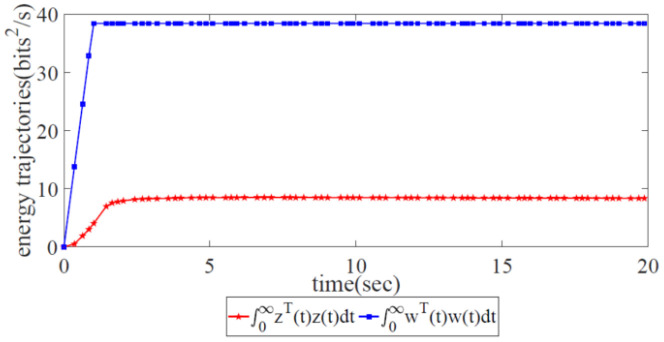
Energy trajectories of the controlled output and the external disturbance under the global robust congestion control scheme.

Figure 8 displays the energy relation between the controlled output z(t) and the external disturbance output w(t). It is notable that the energy trajectory z(t) is lower than w(t), which means that the energy of the external disturbance has been restrained under the robust congestion control scheme. Thus, the effectiveness of our proposed robust congestion control scheme has been validated.

**Figure 9:**
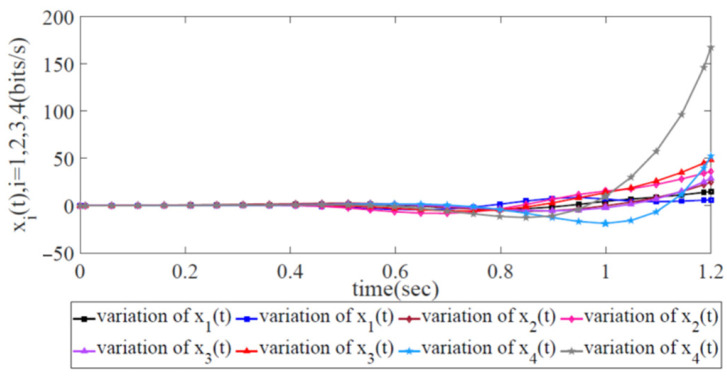
Variation in error state $x_{i}\left(t\right),i=1,2,3,4$ with two trajectories when utilizing the AIMD adjustment scheme.

**Figure 10:**
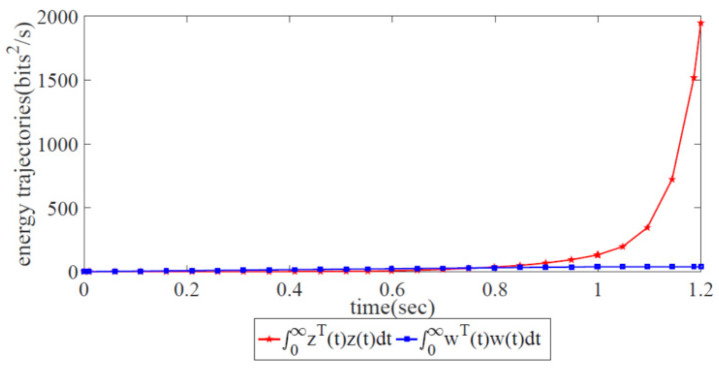
Energy trajectories of the controlled output z(t) and the external disturbance w(t) when utilizing the AIMD adjustment scheme.

**Figure 11:**
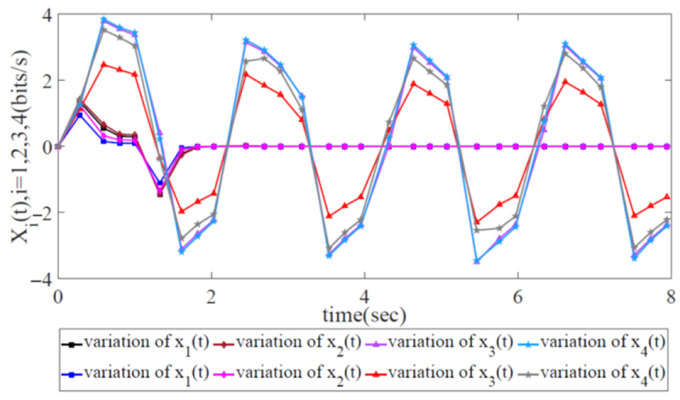
Variation in error state $x_{i}\left(t\right),i=1,2,3,4$ with two trajectories when utilizing the information-forwarding and control algorithm.

**Figure 12:**
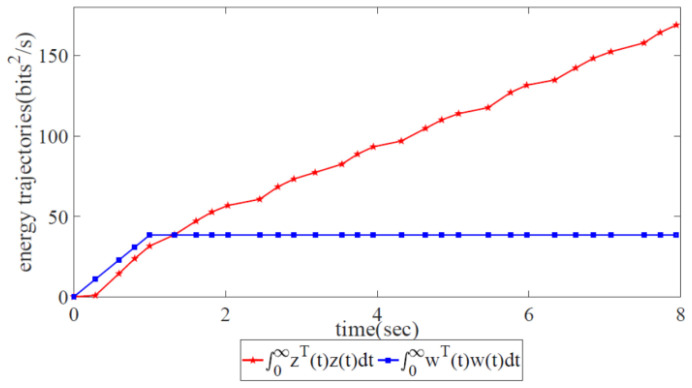
Energy trajectories of the controlled output z(t) and the external disturbance w(t) when utilizing the information-forwarding and control algorithm.

**Figure 13:**
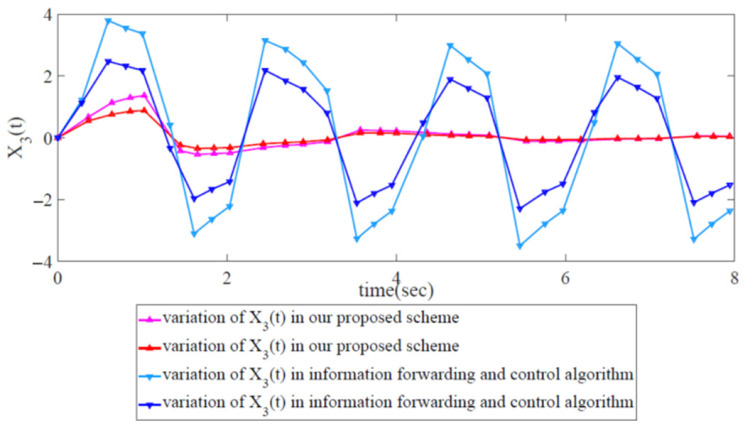
Variation comparison of error states $x_{3}\left(t\right)$ between the information-forwarding and control algorithm and our proposed scheme.

**Figure 14:**
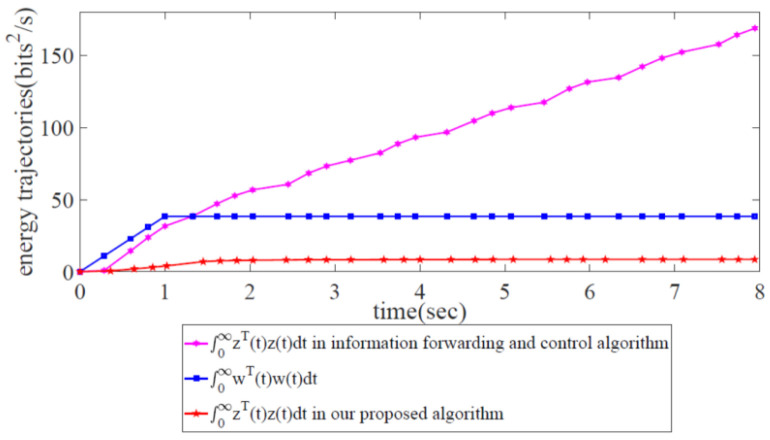
Comparison of energy trajectories between the controlled output z(t) for the information-forwarding and control algorithm, that of our proposed scheme, and external disturbance w(t).

To better reflect the control performance by making simulation-based comparisons with other schemes, including the AIMD adjustment scheme and the information-forwarding and control algorithm, important design elements of the robust H∞ control scheme are ablated to verify the efficiency and robustness of the conditions we determined to be sufficient for achieving global SDWN robustness. The detailed results of the experiments on the robust H∞ control scheme are exhibited in Figure 9, Figure 10, Figure 11, Figure 12, Figure 13 and Figure 14 and listed in Table 1 and Table 2. Table 1 shows the results of the convex optimization after the achievement of global SDWN robustness, in which the iteration number is 60 and the consumption time is 0.0004972 s. It is clear that the convex optimization that implements the weighted fair scheduling strategy for stability congestion control is simple and presents low computational complexity and short time consumption, which satisfies the analysis of Remark. Table 2 shows the results of the robust H∞ control schemes, which include our proposed robust congestion control scheme, the AIMD adjustment scheme, and the information-forwarding and control algorithm. Short discussions comparing the robust H∞ control schemes with the AIMD adjustment scheme and the information-forwarding and control algorithm are presented in the following subsections.

#### 6.3.1. Comparison with AIMD Adjustment Scheme

The error states and energy trajectories corresponding to the traditional AIMD adjustment scheme are shown in Figure 9 and Figure 10, respectively. Obviously, the variations in the error state $x_{i}\left(t\right),i=1,2,3,4$ with two trajectories diverge at 1.2 s in Figure 9. This finding shows that it is impossible for the sending rates of the forwarding devices at the source side to approach the ideal, optimized state. In Figure 10, it is obvious that the energy trajectory z(t) is higher than w(t), i.e., it is impossible for the external disturbance to be restrained under network congestion control. Due to the lack of global information from the SDWN-centralized controllers, the traditional AIMD adjustment scheme cannot satisfy the robustness condition given in Theorem 1.

Upon comparing the traditional AIMD adjustment scheme with our proposed scheme, it can be clearly seen that it is impossible for the sending rates of the forwarding devices at the source side to maintain long-term stability via the traditional AIMD adjustment scheme. This means that the sending rates at the source side diverge from the ideal, optimized states as time passes, which are also incapable of satisfying the demands of global SDWN throughput maximization.

Therefore, this paper demonstrates the superiority of our proposed scheme over the traditional AIMD adjustment scheme in terms of maximizing global SDWN throughput for maintaining long-term stability with two kinds of propagation latencies and external disturbance.

#### 6.3.2. Comparison with Information-Forwarding and Control Algorithm

In terms of the information-forwarding and control algorithm, to solve the network congestion problem, existing works such as [2,34,35,36] emphasize the robust control of partial networks instead of employing a global view of SDWN. This may result in the wireless network failing to achieve global SDWN robustness when the propagation latencies in device–controller pairs are not considered. This paper presents the superiority of our robust congestion control scheme, with two kinds of propagation latency and external disturbance, over the other information-forwarding and control algorithms.

As shown in Figure 11, the variations in error state $x_{i}\left(t\right),i=1,2,3,4$ with two trajectories can indicate network robustness by utilizing the information-forwarding and control algorithm. Clearly, the variations in error state $x_{i}\left(t\right),i=1,2$ correspond to convergence at 1.5 s, while the variations in $x_{i}\left(t\right),i=3,4$ correspond to continuous oscillation (non-convergence). This finding reveals that the information-forwarding and control algorithm for network congestion is suitable for the robust control of partial networks but not for global robust congestion control. Figure 12 shows that the energy trajectory z(t) is notably higher than w(t), which signifies that it is impossible to constrain external disturbance under the network congestion control scheme.

We compared the information-forwarding and control algorithm with our proposed scheme, as shown in Figure 13 and Figure 14. Figure 13 shows the variations in $x_{3}\left(t\right)$ in the case of our proposed scheme and those in the information-forwarding and control algorithm. Obviously, the variations in $x_{3}\left(t\right)$ are under robust control in our proposed scheme, while they are outside of robust control in the information-forwarding and control algorithm. Similarly, in Figure 14, the energy trajectory z(t) in our proposed scheme is also under robust control, while that in the information-forwarding and control algorithm is still outside of robust control.

Therefore, our proposed scheme has been proven to provide more favorable results than the information-forwarding and control algorithm in terms of maximizing the global SDWN throughput for maintaining long-term stability with two kinds of propagation latency and external disturbance.

## 7. Conclusions

In this paper, we have proposed a novel WOA-based robust control scheme, which has been dubbed robust congestion control scheme, with two kinds of propagation latency and external disturbance in SDWNs that has been designed to solve the long-term stabilization problem. First, the sending rate adjustment model was proposed by using the AIMD adjustment scheme with propagation latency in device-to-device paths and the closed-loop congestion control model with propagation latency in device–controller pairs, and the effect of channel competition between neighboring forwarding devices has been analyzed. Next, a robust congestion control model with two kinds of propagation latency and external disturbance was established. Then, an efficient WOA-based scheduling strategy was presented in order maximize global network throughput. Afterward, the sufficient conditions were derived using Lyapunov–Krasovskii functionals and formulated by LMIs. Finally, after comparing the traditional AIMD adjustment scheme with an existing information-forwarding and control algorithm, we conducted numerical network simulations to demonstrate the effectiveness of our robust congestion control scheme. This scheme could also be utilized to analyze other efficient robust control methods in SDWNs. For future research, more applicable control algorithms modeled in SDWNs could be discussed in order to improve reliability and scalability. Simultaneously, more efficient SDN scheduling strategies could be further studied and improved.

## Figures and Tables

**Table 1:** The results for the WOA algorithm.

	WOA Algorithm
Iteration number	60
Consumption time (sec)	0.0004972

**Table 2:** The results for the robust H∞ control schemes.

Schemes	Characteristics	Convergence	Energy Trajectories
Our proposed robust congestion control scheme	Two kinds of propagation latency and channel competition	Convergence	J<0
AIMD adjustment scheme	Propagation latency in device-to-device paths	Non-convergence	J>0
Information-forwarding and control algorithm	Propagation latency in device-to-device paths and channel competition	Non-convergence	J>0
